# Establishment of a Pig Influenza Challenge Model for Evaluation of Monoclonal Antibody Delivery Platforms

**DOI:** 10.4049/jimmunol.2000429

**Published:** 2020-07-17

**Authors:** Adam McNee, Trevor R. F. Smith, Barbara Holzer, Becky Clark, Emily Bessell, Ghiabe Guibinga, Heather Brown, Katherine Schultheis, Paul Fisher, Stephanie Ramos, Alejandro Nunez, Matthieu Bernard, Simon Graham, Veronica Martini, Tiphany Chrun, Yongli Xiao, John C. Kash, Jeffery K. Taubenberger, Sarah Elliott, Ami Patel, Peter Beverley, Pramila Rijal, David B. Weiner, Alain Townsend, Kate E. Broderick, Elma Tchilian

**Affiliations:** * The Pirbright Institute, Pirbright GU24 0NF, United Kingdom;; † Inovio Pharmaceuticals, San Diego, CA 92121;; ‡ Animal and Plant Health Agency-Weybridge, New Haw, Addlestone KT15 3NB, United Kingdom;; § Viral Pathogenesis and Evolution Section, Laboratory of Infectious Diseases, National Institute of Allergy and Infectious Diseases, National Institutes of Health, Bethesda, MD 20892-3203;; ¶ Vaccine and Immunotherapy Center, The Wistar Institute, Philadelphia, PA 19103;; ‖ National Heart and Lung Institute, St Mary’s Campus, Imperial College London, London W2 1PG, United Kingdom; and; # Weatherall Institute of Molecular Medicine, University of Oxford, Oxford OX3 9DS, United Kingdom

## Abstract

mAbs are a possible adjunct to vaccination and drugs in treatment of influenza virus infection. However, questions remain whether small animal models accurately predict efficacy in humans. We have established the pig, a large natural host animal for influenza, with many physiological similarities to humans, as a robust model for testing mAbs. We show that a strongly neutralizing mAb (2–12C) against the hemagglutinin head administered prophylactically at 15 mg/kg reduced viral load and lung pathology after pandemic H1N1 influenza challenge. A lower dose of 1 mg/kg of 2–12C or a DNA plasmid–encoded version of 2–12C reduced pathology and viral load in the lungs but not viral shedding in nasal swabs. We propose that the pig influenza model will be useful for testing candidate mAbs and emerging delivery platforms prior to human trials.

## Key Points

- Neutralizing mAb 2–12C reduces influenza viral load and lung pathology in pigs.
- DNA plasmid–encoded 2–12C reduces lung pathology.
- The pig is a useful preclinical model for testing mAbs and mAb delivery platforms.

## Introduction

Influenza virus infection remains a significant global health threat to humans and livestock, causing substantial mortality and morbidity. mAbs administered either prophylactically or therapeutically have been proposed as a strategy to provide immediate immunity and augment existing vaccines and drugs in combatting seasonal and pandemic influenza infection. Broadly neutralizing Abs against conserved epitopes of the hemagglutinin (HA) stem and head and Abs against the neuraminidase (NA) are candidates for human treatment (1, 2). Both prophylactic and therapeutic administration of these Abs have been shown to be effective in the mouse and ferret (3–10). However, early results from human clinical trials showed that efficacy in mice and ferrets is not always predictive of outcome in humans (11–14). The reasons for the apparent lack of efficacy in humans are not clear but may include the difficulty of achieving high serum and nasal concentration in a large body mass, the potency of the mAbs, or the challenge of therapeutic administration in the face of a high viral load. Variability due to pre-existing immunity in human experimental or natural infection challenge studies is an additional problem. There is, therefore, a need for a large animal model in which mAbs selected on the basis of in vitro assays and efficacy in small animals can be further studied to help in selecting promising mAbs and determining how best to administer them in clinical trials. Pigs may provide such a model. They are large animals and a natural host for influenza viruses. Pigs and humans are infected by the same subtypes of virus, have the same distribution of sialic acid receptors in their respiratory tract, and are physiologically, anatomically, and immunologically more similar to humans than small animals (15, 16).

Although great progress in Ab delivery is being made, the high costs that are associated with the production, purification, and quality control are major challenges in the development of clinical mAbs against influenza and other infectious diseases. Moreover, long-term protection is difficult with a single inoculation because of the short half-life of the mAb. Alternative in vivo Ab gene transfer strategies using DNA, RNA, or viral vectors have shown that Ab genes can be stably maintained in the host tissue, resulting in potent and long-term expression of mAbs in the body following a single administration (17–23). DNA plasmid–encoded mAbs (dMAbs), which are delivered to muscle tissue, are a novel approach with the potential to provide durable immunity (24–26). The plasmid DNA is well tolerated and nonintegrating, does not require cold-chain distribution, can be delivered repeatedly, and is relatively inexpensive to produce. Previous studies have demonstrated the efficacy of such an approach for protection against influenza in mice (27).

We have previously tested therapeutic administration of the broadly neutralizing anti-stem FI6 Ab in the pig influenza model. This did not reduce viral load in nasal swabs and bronchoalveolar lavage (BAL), although there was reduction of pathology after aerosol delivery (28). Broadly neutralizing anti-stem mAbs are less potent at direct viral neutralization as compared with anti-head Abs and require Fc receptor engagement for in vivo protection (29, 30). We demonstrated that human IgG1 FI6 did not bind to pig Fc receptors, perhaps accounting for the weak effect. Therefore, to establish a more robust pig model, we reasoned that a strongly neutralizing strain-specific anti-head HA mAb should overcome this problem and give clear protection, providing a benchmark against which other mAbs and delivery platforms might be tested. In this study we used the 2–12C mAb isolated from an H1N1pdm09-exposed individual, which shows strong neutralizing activity and selects influenza virus variants with HA substitutions K130E (31). Furthermore, we administered 2–12C prophylactically to provide the best chance to reveal an effect on viral load, as it is challenging for therapeutic administration to reduce viral load postinfection after it has been established. We went on to evaluate the potential of an in vivo–produced 2–12C using synthetic dMAb technology in support of the translation of this technology to humans as an immunoprophylactic.

## Materials and Methods

### Ab preparation

The anti-influenza human IgG1 mAb 2–12C and the antifluorescein human IgG1 isotype control were produced in bulk by Absolute Ab Ltd (Redcar, U.K.). They were dissolved in 25 mM histidine, 150 mM NaCl, and 0.02% Tween P80 (pH 6) diluent. We also engineered synthetic plasmid DNA to encode the human 2–12C. A single plasmid was designed to encode both the mAb 2–12C HC and LCs under the control of a human CMV promoter and human IgG signal sequence and bovine growth hormone polyadenylation signal. The heavy and light Ig chains genes were separated by both a furin cleavage and a P2A cleavage site to ensure complete processing of the two proteins. The resulting construct was named dMAb 2–12C.

### Influenza challenge studies in pigs

All experiments were approved by the ethical review processes at the Pirbright Institute and Animal and Plant Health Agency (APHA) and conducted according to the U.K. Government Animal (Scientific Procedures) Act 1986. APHA conforms to Animal Research: Reporting In Vivo Experiments guidelines. Two pig influenza virus challenge experiments were carried out.

For the first experiment, 15 5-wk-old Landrace × Hampshire cross, female pigs were obtained from a commercial high-health status herd and were screened for absence of influenza A infection by matrix gene real time RT-PCR and for Ab-free status by hemagglutination inhibition using four swine influenza virus Ags: pdmH1N1, H1N2, H3N2, and avian-like H1N1. Pigs weighed between 11 and 14 kg (average 12.5 kg). Pigs were randomized (https://www.graphpad.com/quickcalcs/randomize1/) into three groups of five animals as follows: the first control group received 1 ml/kg histidine diluent only; the second isotype control group received 15 mg/kg antifluorescein IgG1 mAb i.v.; and the third 2–12C group received 15 mg/kg 2–12C i.v. The Abs were administered to the ear vein of animals sedated with stresnil. Twenty-four hours after mAb administration, all animals were challenged intranasally with 3 × 10^6^ PFUs of pandemic swine H1N1 isolate, A/swine/England/1353/2009 (pH1N1) in 4 ml (2 ml per nostril) using a mucosal atomization device (MAD300; Wolfe Tory Medical).

In the second challenge experiment, 30 5-wk-old Landrace × Hampshire cross, influenza virus–free female pigs were obtained, and their influenza-free status was confirmed as above. The weights of the pigs were between 12 and 14 kg (average of 12.9 kg). The pigs were randomized into four groups of six animals as follows: control went untreated, 15 mg/kg recombinant 2–12C i.v., 1 mg/kg recombinant 2–12C i.v., and the final group received 6 mg of human dMAb 2–12C. Recombinant 2–12C protein was administered i.v. 24 h before the pH1N1 challenge to sedated pigs, and dMAbs were administered by electroporation (EP) to pigs sedated with 4 mg/kg Zoletil and 0.04 mg/kg Domitor 6 d before the pH1N1 influenza virus challenge. Six milligrams of dMAb 2–12C plasmid DNA (pDNA) was formulated with 135 U/ml Hylenex (Halozyme Therapeutics, San Diego CA), the formulation was administered i.m. to the back-left quadriceps of the animal, and followed by in vivo EP using the CELLECTRA constant current device. All animals were challenged with 3.4 × 10^6^ PFUs of pH1N1 in 4 ml (2 ml per nostril) using a mucosal atomization device. Clinical signs (temperature, state of breathing, coughing, nasal discharge, appetite, altered behavior) observed were mild and none of the pigs developed moderate or severe disease.

### Pathological and histopathological examination of lungs

Animals were humanely killed 4 d postinfection (DPI) with an overdose of pentobarbital sodium anesthetic. The lungs were removed, and digital photographs taken of the dorsal and ventral aspects. Macroscopic pathology was scored blind as previously reported (32). The percentage of the lung displaying gross lesions for each animal was calculated using image analysis software (Fiji ImageJ) on the digital photographs. Lung tissue samples from cranial, middle, and caudal lung lobes were taken from the left lung and collected into 10% neutral-buffered formalin for routine histological processing. Formalin-fixed tissues were paraffin wax–embedded, and 4-μm sections were cut and routinely stained with H&E. Immunohistochemical detection of influenza A virus nucleoprotein (NP) was performed in 4-μm tissue sections as previously described (33). Histopathological changes in the stained lung tissue sections were scored by a veterinary pathologist blinded to the treatment group. Lung histopathology was scored using five parameters (necrosis of the bronchiolar epithelium, airway inflammation, perivascular/bronchiolar cuffing, alveolar exudates, and septal inflammation) scored on a five-point scale of 0–4 and then summed to give a total slide score ranging from 0–20 per slide and a total animal score from 0–60 (34). The slides were also scored using the “Iowa” method, which also takes into account the amount of viral Ag present in the sample, as described (35).

### Tissue sample processing

Four nasal swabs (two per nostril) were taken at 0, 1, 2, 3, 4 DPI. The swabs were placed into 1 ml of TRIzol or 2 ml of virus transport medium comprising tissue culture medium 199 (Sigma-Aldrich, St. Louis, MO) supplemented with 25 mM HEPES, 0.035% sodium bicarbonate, 0.5% BSA, 100 IU/ml penicillin, 100 μg/ml streptomycin, and 0.25 μg/ml nystatin, vortexed, centrifuged to remove debris, and stored at −80°C for subsequent virus titration. Blood samples were collected at the start of the study (prior to Ab administration) and at the indicated times post-mAb delivery and challenge. BAL was collected from the entire left lung with 150 ml of virus transport medium (described above). BAL samples were centrifuged at 300 × *g* for 15 min and the supernatant was removed, aliquoted, and frozen for Ab analysis.

### Virus titration and viral RNA isolation

Viral titers in nasal swabs, BAL, and accessory lung lobe were determined by plaque assay on Madin–Darby canine kidney (MDCK) cells (Central Service Unit, The Pirbright Institute). Samples were 10-fold serially diluted in DMEM, and 100 μl were overlaid on confluent MDCK cells in 12-well tissue culture plates. After 1 h, the plates were washed and overlaid with 2 ml of 0.66% agarose containing culture medium. Plates were incubated at 37°C for 48–72 h, and plaques were visualized using 0.1% crystal violet. For sequencing, nasal swabs were collected in 1 ml of TRIzol (Invitrogen, Thermo Fisher Scientific, Waltham, MA), and RNA was extracted by chloroform and isopropanol precipitation.

### Next generation sequencing

Viral RNA–enriched library production and viral cDNA library sequencing were as previously reported (36). Briefly, isolated RNA was amplified using the Ovation RNA-Seq System V2 from NuGEN (NuGEN, San Carlos, CA). The amplified total cDNAs were analyzed by an Agilent 2100 Bioanalyzer using the Agilent High Sensitivity DNA Kit (Agilent Technologies, Santa Clara, CA) and sheared to 150 bp on the Covaris S2 Machine (Covaris, Woburn, MA). Approximately 400 ng of amplified cDNA was used to generate the Illumina sequencing library using the Agilent SureSelectXT Target Enrichment Kit (Agilent) for Illumina multiplex sequencing by using enrichment probes designed for A/California/04/2009(H1N1) virus. Enriched Illumina sequencing libraries were sequenced on an Illumina NextSeq Sequencer (Illumina, San Diego, CA).

### Data analysis

Reads were mapped to the HISAT2-indexed A/swine/England/1353/2009 (pH1N1) genome using the alignment program for mapping next generation sequencing reads HISAT2 (release 2.0.5, https://ccb.jhu.edu/software/hisat2/index.shtml) downloaded from the Center for Computational Biology, Johns Hopkins University (37). SAMtools mpileup (version 2.1.0) (38) was used to make single-nucleotide polymorphism (SNP) calls with the minimum base Phred quality score as 25. A reported SNP call was one that satisfied the following criteria at the SNP position: 1) more than 100 reads at that position (39–41), 2) reads present from both directions, 3) variant calls exactly at the end of the read eliminated, and 4) reads with bases that are different to reference more than 10% of the aligned reads.

### Microneutralization assay

Neutralizing Ab titers were determined in serum and BAL fluid using a microneutralization (MN) assay as previously described (42). In brief, pig sera or BAL fluid were heat-treated for 30 min at 56°C and diluted 1:10 for serum and 1:2 for BAL as a starting point for the assay. Fifty microliters of serially diluted samples were incubated with an equal volume of pH1N1 (the virus was titrated beforehand in the absence of serum to determine the PFU/ml necessary to yield a plateau infection in the MN assay). After 2 h, MDCK SIAT-1 cells at 3 × 10^4^ cells per well were added to the serum/virus and incubated for 18 h. The fixed and permeabilized cell monolayer was stained with anti-NP (clone: AA5H; Bio-Rad Laboratories) followed by goat anti-mouse HRP (Dako) Ab. After addition of the 3,3′,5,5′-tetramethylbenzidine (TMB) substrate, the reaction was stopped with 1 M sulfuric acid, and absorbance was measured at 450 and 570 nm (reference wavelength) on the Cytation3 Imaging Reader (Biotek Instruments). The MN titers were expressed as half maximal inhibitory dilution (50% inhibitory titer is the midpoint between uninfected control wells and virus-infected positive controls) derived by linear interpolation from neighboring points in the titration curve.

### ELISAs

Ab titers against the pH1N1 HA in the serum, BAL fluid, and nasal swabs were determined by ELISA. The recombinant HA protein of A/Eng/195/2009 containing a C-terminal thrombin cleavage site, a trimerization sequence, a hexahistidine tag, and a BirA recognition sequence was expressed in HEK293 cells and purified as described previously (42). Ninety-six–well microtiter plates (Maxi Sorp, Nunc; Sigma-Aldrich) were coated with 50 μl of recombinant protein at a concentration of 1 μg/ml in carbonate buffer overnight at 4°C. Plates were blocked with 200 μl of blocking solution of 4% milk powder in PBS supplemented with 0.05% Tween-20 (PBS-T) for 2 h at room temperature. Samples were serially diluted in PBS-T with 4% milk powder and added to the wells for 1 h on a rocking platform. The plates were washed three times with PBS-T, 100 μl of HRP-conjugated goat anti-human or goat anti-pig Fc fragment secondary Ab (Bethyl Laboratories) diluted in PBS-T with 4% milk powder was added, and plates were incubated for 1 h at room temperature. The plates were washed four times with PBS-T and developed with 100 μl per well TMB High Sensitivity Substrate Solution (BioLegend). After 5–10 min, the reaction was stopped with 100 μl of 1 M sulfuric acid, and the plates were read at 450 and 570 nm with the Cytation3 Imaging Reader (Biotek Instruments). A standard curve was generated using recombinant 2–12C Ab (Absolute Ab). The data were analyzed in Microsoft Excel and GraphPad Prism. The cut off value was defined as the average of all blank wells plus three times the SD of the blank wells.

For detection of an Ab response to 2–12C in pigs (anti-drug Ab [ADA] response), 96-well microtiter plates (Maxi Sorp, Nunc; Sigma-Aldrich) were coated with 2 μg/ml recombinant 2–12C (Absolute Ab) in PBS-T overnight at 4°C. The plates were washed and blocked. Prediluted serum samples in PBS-T were added and incubated for 2 h at room temperature. The plates were washed three times, and goat anti-pig H + L chain–HRP (Bethyl Laboratories) was added for 1 h at room temperature and developed as above.

### In vitro transfection of dMAb 2–12C

Lipofectamine 3000 Transfection Kit (Thermo Fisher Scientific) was used to transfect adherent HEK 293T cells (ATCC CRL11268) with dAMb 2–12C plasmids. Medium was harvested 72 h posttransfection and filtered using 0.22-μm Stericup-GP Vacuum Filtration System (Millipore, Burlington, MA). The supernatant from pDNA-transfected cells was purified using Protein G GraviTrap (GE Healthcare, Chicago, IL) according to the manufacturer’s instructions. The eluted protein was concentrated by Amicon Ultra-15 Centrifugal Filter Unit (30 kDa) and quantified by nanodrop.

### Western blot

A total of 0.5 μg of each sample was loaded on a NuPAGE 4–12% Bis-Tris gel (Thermo Fisher Scientific). Precision Plus Protein Kaleidoscope Prestained Protein Standard (Bio-Rad Laboratories, Hercules, CA) was used as the standard marker. The gel was transferred to a polyvinylidene difluoride membrane using an iBlot 2 Transfer device (Invitrogen). The membrane was blocked with goat histology buffer (1% BSA [Sigma-Aldrich], 2% goat serum [Sigma-Aldrich], 0.3% Triton-X [Sigma-Aldrich], and 0.025% 1g/ml sodium azide [Sigma-Aldrich] in PBS) for 30 min at room temperature. Goat anti-human IgG-Fc fragment Ab (A80-104A; Bethyl Laboratories, Montgomery, TX) diluted in 1:1000 in goat histology buffer was added and incubated for 1 h at room temperature. After washing the blot for 5 min in Dulbecco PBS (DPBS) (HyClone, Logan, UT) three times, donkey anti-goat IgG HRP Ab (Abcam, Cambridge, U.K.) in 1:2000 dilution in goat histology buffer was added and incubated for 1 h at room temperature. After washing the blot three times for 5 min in DPBS, the membrane was developed using the ECL Prime Western blotting System (GE Healthcare) and imaged using the Protein Simple FluorChem System.

### In vivo dMAb 2–12C mouse and pig immunogenicity and pharmacokinetic studies

Female BALB/c mice between 4 and 6 wk of age were group-housed with ad libitum access to feed and water. Husbandry was provided by Acculab (San Diego, CA), and all procedures were in compliance with the standards and protocols of the Institutional Animal Care and Use Committee at Acculab. To deplete T cell populations, BALB/c mice received one i.p. injection of 500 μg of anti-mouse CD4 (BE0003-1; BioXCell, West Lebanon, NH) and 500 μg of anti-mouse CD8α (BE0117; BioXCell) in 300 μl of PBS. Two hundred micrograms dMAb plasmid DNA was formulated with 135 U/ml of human recombinant hyaluronidase (Hylenex; Halozyme Therapeutics, San Diego, CA). Mice received an i.m. administration of formulation followed by EP. EP was delivered at the injection site with the CELLECTRA-3P Adaptive In Vivo Electroporation System. An array of three-needle electrodes with a 3-mm insertion depth was used. The EP treatment consists of two sets of pulses with 0.1 Amp constant current with the second pulse set delayed by 3 s. Within each set there are two 52-ms pulses with a 198-ms delay between the pulses. One hundred microliters of recombinant 2–12C was injected i.v. at a dose of 1 mg/kg.

Pigs received i.m. administration of up to 24 mg of dMAb plasmid DNA formulated with 135 U/ml of human recombinant hyaluronidase. EP was delivered at the injection site with the CELLECTRA-5P Adaptive In Vivo Electroporation System. For pharmacokinetic studies, blood samples were drawn at the indicated time points.

### Immunofluorescence staining and imaging

In vivo–expressed 2–12C 3 d after i.m. delivery of dMAb 2–12C pDNA, mouse and pig muscle tissues were harvested, fixed in 10% neutral-buffered formalin (BBC Biochemical, Stanford, MA) and immersed in 30% sucrose (Sigma-Aldrich) in deionized water for in vivo staining of dMAb expression. Tissues were then embedded into O.C.T. compound (Sakura Finetek, Torrance, CA) and snap-frozen. Frozen tissue blocks were sectioned to a thickness of 18 μm. Slides were incubated with blocking buffer (0.3% Triton-X [Sigma-Aldrich], 2% donkey serum in PBS) for 30 min, covered with parafilm. Goat anti-human IgG-Fc Ab (Bethyl Laboratories) was diluted 1:100 in incubation buffer (1% BSA [Sigma-Aldrich], 2% donkey serum, 0.3% Triton-X [Sigma-Aldrich], and 0.025% 1 g/ml sodium azide [Sigma-Aldrich] in PBS). One hundred fifty microliters of staining solution was added to each slide and incubated for 2 h. Slides were washed in PBS three times. Donkey anti-goat IgG AF488 (Abcam) was diluted 1:200 in incubation buffer, and 50 μl was added to each section. Slides were washed after 1 h incubation and mounted with DAPI-Fluoromount (SouthernBiotech, Birmingham, AL) and covered. Slides were imaged with a BX51 Fluorescent Microscope (Olympus, Center Valley, PA) equipped with Retiga3000 Monochromatic Camera (QImaging, Surrey, Canada).

### Quantification of human IgG in dMAb 2–12C-treated animals

Ninety-six–well assay plates (Thermo Fisher Scientific) were coated with 1 μg per well goat anti-human IgG-Fc fragment Ab (Bethyl Laboratories) in DPBS (Thermo Fisher Scientific) overnight at 4°C. The next day, plates were washed with 0.2% Tween-20 in PBS and blocked with 10% FBS in DPBS for 1 h at room temperature. The serum samples were diluted in 1% FBS in 0.2% Tween-PBS, and 100 μl of this mix was added to the washed assay plate. Additionally, a standard curve of recombinant 2–12C mAb prepared as 1:2 serial dilutions starting at 500 ng/ml in dilution buffer and added in duplicate to each assay plate. Samples and standard were incubated for 1 h at room temperature. After washing, the plates were incubated with a 1:10,000 dilution of goat anti-human IgG-Fc Ab HRP (A80-104P; Bethyl Laboratories) for 1 h at room temperature. For detection, SureBlue Substrate Solution (SeraCare Life Sciences, Milford, MA) was added to the washed plates. The reaction was stopped by adding TMB Stop Solution (SeraCare Life Sciences) after 6 min to the assay plates. The ODs were read at 450 nm. The serum concentration was interpolated from the standard curve using a sigmoidal four-parameter logistic curve fit for log of the concentration.

### Statistical analysis

One-way nonparametric ANOVA (Kruskal–Wallis) with Dunn posttest for multiple comparisons was performed using GraphPad Prism 8.3.

## Results

### Establishment of a prophylactic pig influenza challenge model with recombinant 2–12C mAb

**FIGURE 1.**
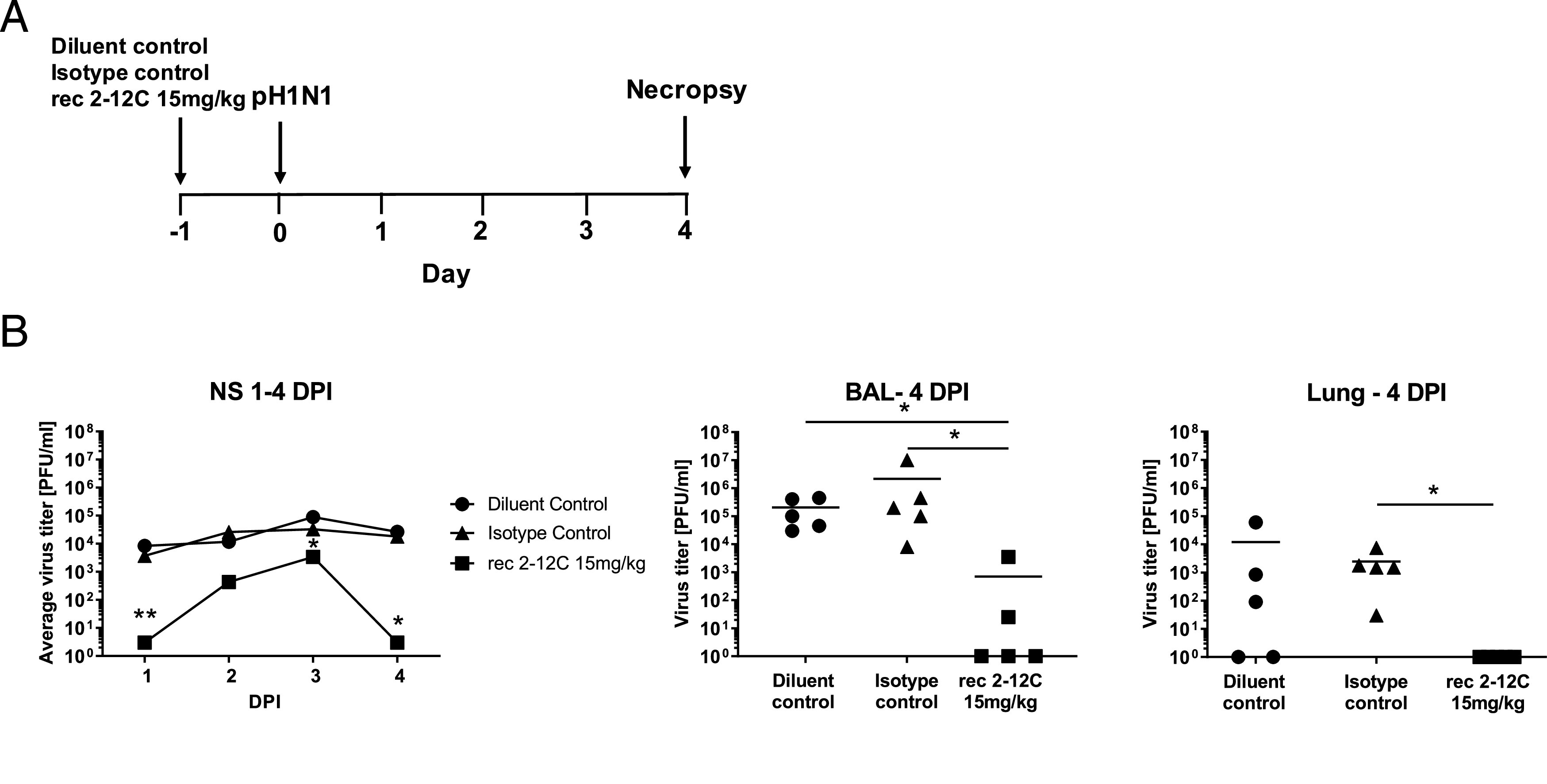
Experimental design and viral load. Abs or diluent were administered i.v. to pigs, which were infected with pH1N1 virus 24 h later. Nasal swabs (NS) were taken at 1, 2, 3, and 4 DPI, and pigs sacrificed at 4 DPI (**A**). Viral titers in nasal swabs, accessory lung lobe (Lung), and BAL were determined by plaque assay (**B**). Viral shedding in NS is represented as the mean of the five pigs on each day and the significance versus diluent control indicated by asterisks. Each data point in BAL and lung represents an individual pig, and bars show the mean. Viral titers were analyzed using one-way nonparametric ANOVA, the Kruskal–Wallis test. Asterisks denote significant differences *p < 0.05, **p < 0.01, versus indicated control groups.

To establish a positive control protective mAb and delivery method in the pig influenza model, we tested the strongly neutralizing human IgG1 anti-head 2–12C mAb (31) in a prophylactic experiment. We administered 15 mg/kg of 2–12C i.v. and the control groups received an isotype-matched IgG1 mAb or the diluent. Twenty-four hours later, the pigs were challenged with pandemic swine H1N1 isolate, A/swine/England/1353/2009 (pH1N1) and, 4 d later, culled to assess viral load and pathology (Fig. 1A). The 2–12C significantly decreased viral load in nasals swabs on each day over the course of infection, although the decrease was less on days 2 and 3 than days 1 and 4 (Fig. 1B). However, because the total viral load in nasal swabs over the 4 d in animals treated with 2–12C was also significantly less as determined by the area under the curve compared with diluent and isotype controls (*p* = 0.0267 and *p* = 0.021), viral load in the 2–12C group was significantly reduced in the BAL, and no virus was detected in the lungs at 4 DPI. Overall, 2–12C had a clear effect on viral load in nasal swabs, BAL, and lung.

**FIGURE 2.**
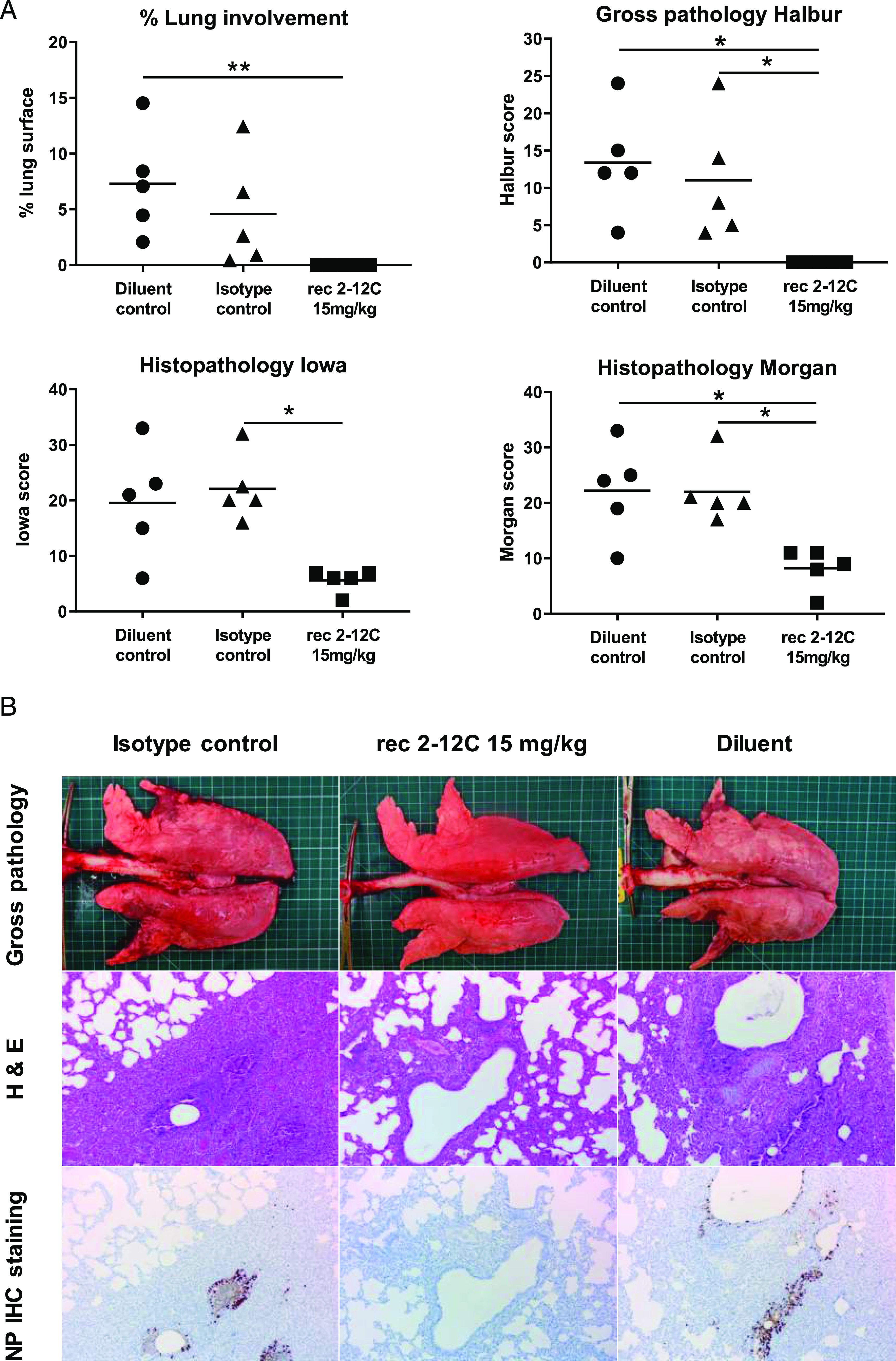
Lung pathology. Abs or diluent were administered i.v. to pigs, which were infected with pH1N1 virus 24 h later. The animals were culled at 4 DPI, and lungs were scored for appearance of gross and histopathological lesions. The score for each individual in a group and the group means are shown (**A**). Representative gross pathology, histopathology (H&E staining; original magnification ×100), and immunohistochemical NP staining (original magnification ×200) for each group are shown (**B**). Pathology scores were analyzed using one-way nonparametric ANOVA with the Kruskal–Wallis test. Asterisks denote significant differences *p < 0.05, **p < 0.01, versus indicated control groups.

The isotype and diluent control groups showed the most severe gross pathology and histopathology scores (Fig. 2A). The red tan areas of pulmonary consolidation were present mainly in the cranial and middle lung lobes of animals from the isotype and diluent groups, but no lesions were found in the 2–12C group (Fig. 2B). Similarly, necrotizing bronchiolitis and bronchial and alveolar exudation, and mild thickening of the alveolar septa, was shown in the animals from both control groups. Minimal to mild alveolar septa thickening and lymphoplasmacytic peribronchial infiltration was present in only one animal from the 2–12C-treated group. Influenza A NP (brown labeling) was detected by immunohistochemistry (IHC) in the bronchial and bronchiolar epithelial cells, alveolar cells, and exudate in the bronchioles and alveoli in both control groups. No NP labeling was found in the lung of animals treated with 2–12C.

**FIGURE 3.**
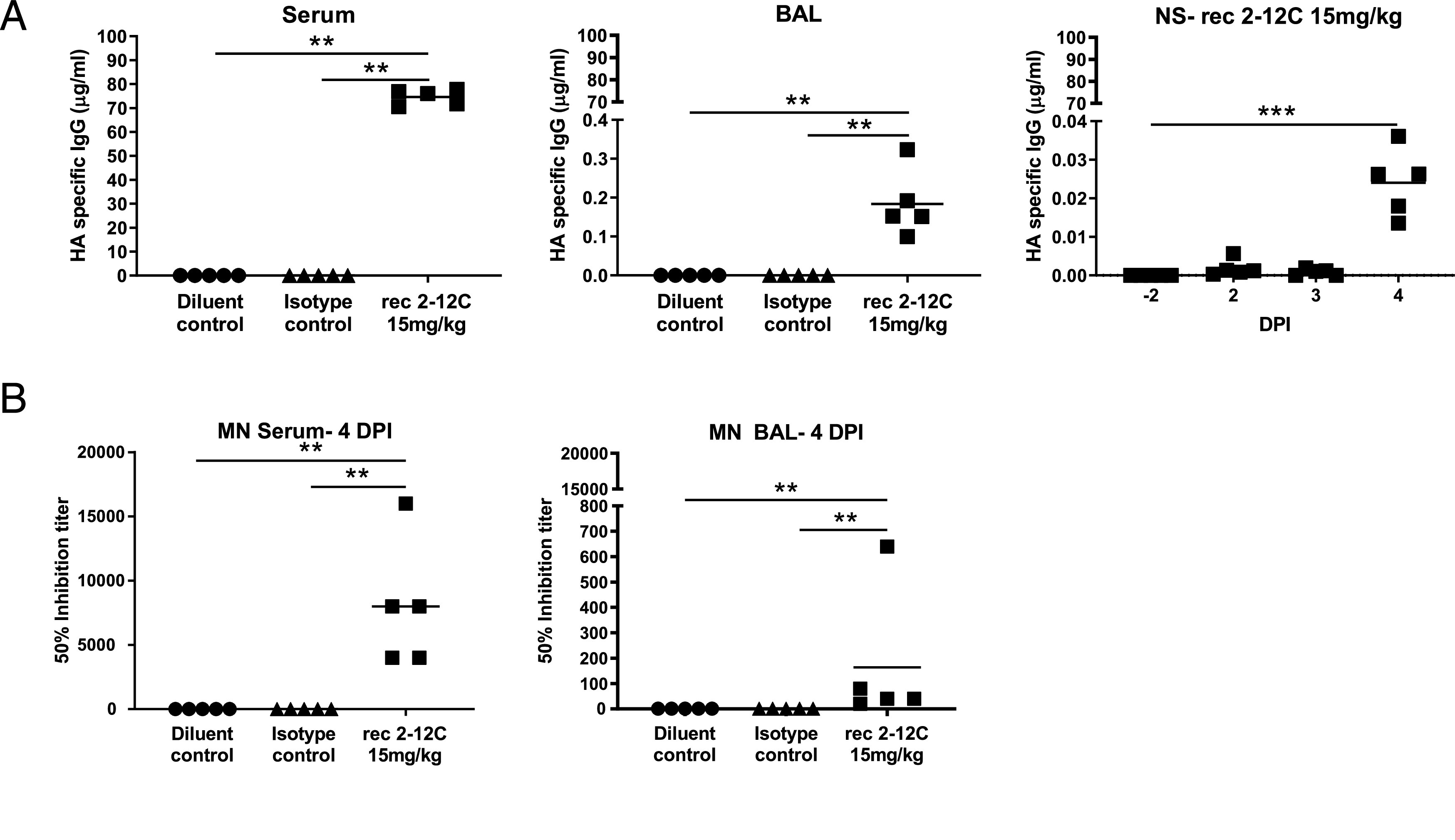
Concentration and neutralizing titer of 2–12C in serum and mucosal tissues. H1 HA-specific IgG in serum, BAL at 4 DPI, and nasal swabs (NS) at the indicated DPI (**A**). A total of 50% neutralization titers against pH1N1 in the serum and BAL at 4 DPI (**B**). Symbols represent an individual pig within the indicated group, and lines represent the mean. Data were analyzed using one-way nonparametric ANOVA with the Kruskal–Wallis test. Asterisks denote significant differences **p < 0.01, ***p < 0.001, versus indicated control groups.

Recombinant 2–12C was detected in the serum at a mean concentration of 74.6 μg/ml and in the BAL at 183.9 ng/ml at 4 DPI as determined by an HA ELISA (Fig. 3A). HA-specific IgG was also detected in nasal swabs at a mean concentration of 24 ng/ml. The serum of the 2–12C-treated group exhibited strong neutralizing activity at a 50% inhibition titer of 1:8000 and 1:164 in the BAL (Fig. 3B). Neutralizing activity in nasal swabs could not be tested because of the limited sample availability. These data show that prophylactic i.v. administration of recombinant 2–12C at 15 mg/kg significantly reduced viral load and pathology in the pig, a large natural host animal.

### Design and expression of dMAb in pigs and mice

**FIGURE 4.**
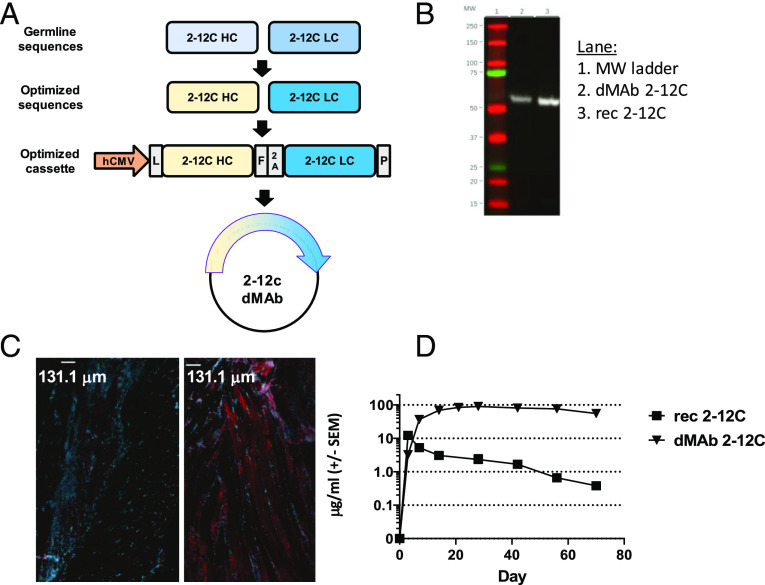
Design and expression of anti-influenza dMAb 2–12C. dMAb 2–12C single construct was designed to express human Ig LC and HC sequence codons and were RNA optimized for in vivo expression (**A**). The optimized cassette includes a human CMV promoter (hCMV), a human IgG signal sequence (L), 2–12C HC and LC separated by a furin (F) and P2A cleavage site, and bovine growth hormone polyadenylation signal (P). In vitro expression of dMab 2–12C (**B**). Supernatant from dMAb 2–12C plasmid–transfected 293 T cells was run on a Western blot next to recombinant 2–12C mAb using a detection Ab reactive to human IgG HC. In vivo expression of dMAb 2–12C (**C** and **D**). dMAb 2–12C or pVAX pDNA was administered i.m. by EP to BALB/c mice. Immunofluorescence images were taken on muscle tissue sections harvested 3 d after pDNA administration (C). Scale bars, 131.1 μm. Blue denotes DAPI; red denotes human IgG. Serum human IgG levels after day 0 administration of recombinant or dMAb 2–12C in BALB/c mice quantified by ELISA (D).

To investigate the potential of a DNA-launched influenza mAb to mediate disease protection in the pigs, we designed and engineered dMAb 2–12C (Fig. 4A). The gene sequences of the human IgG1 HC and LCs were codon- and RNA optimized and inserted into a single modified pVax1 DNA expression vector plasmid, separated by furin and P2A peptide cleavage sites. Expression of dMAb 2–12C was confirmed by Western blot analysis, the band was detected at the same m.w. as recombinant 2–12C (Fig. 4B). To assess in vivo expression, dMAb 2–12C was formulated with human recombinant hyaluronidase, as an optimization to enhance gene expression in the context of delivery with adaptive in vivo EP (43). We measured expression of human IgG in myocytes 3 d after the administration of dMAb 2–12C pDNA into the tibialis anterior muscle of BALB/c mice, confirming local expression of human IgG at the site of delivery (Fig. 4C). To assess levels and durability of in vivo expression, mice were conditioned to reduce the T cell compartment using CD4- and CD8-depleting Abs; this has been shown to permit the expression of a human mAb construct in mice in the absence of a host ADA response (25). Administration of dMAb 2–12C pDNA to the tibialis anterior muscle was associated an increased durability of circulating levels of the mAb in the serum compared with recombinant 2–12C, administered i.v. (Fig. 4D).

### In vivo expression of dMAb 2–12C was measured in pigs

**FIGURE 5.**
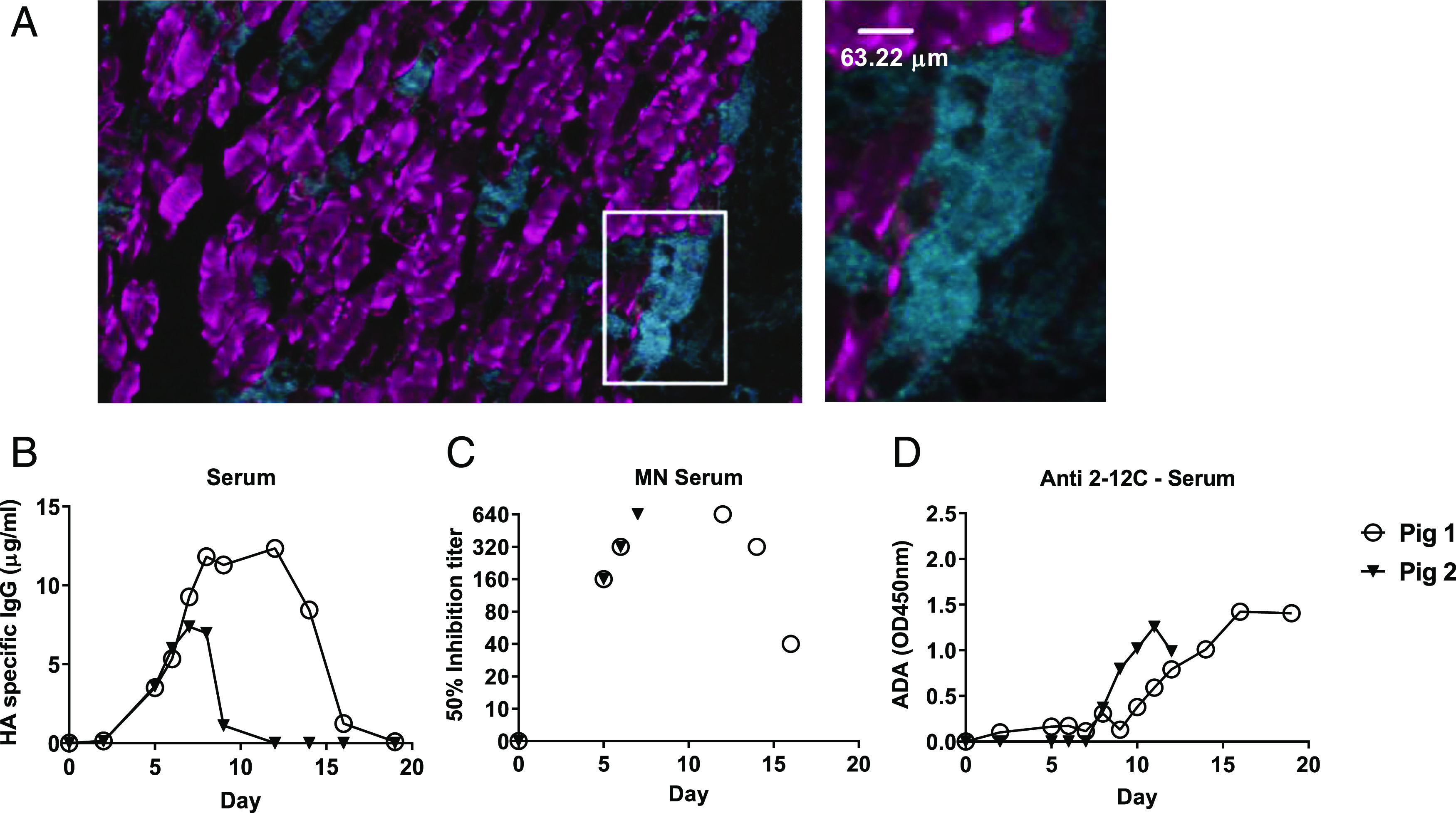
Expression of dMAb 2–12C in pigs. dMAb 2–12C pDNA was administered into the quadricep muscles of Yorkshire pigs on day 0. Immunofluorescence images were taken on muscle tissue sections harvested 3 d after pDNA administration. The outlined area is shown magnified to the right (**A**). Scale bar, 63.22 μm. Blue denotes DAPI; red denotes human IgG. Serum 2–12C levels after day 0 administration of dMAb 2–12C were quantified by ELISA (**B**). Serum neutralizing activity of H1N1 in an MN assay (**C**). ADA response against 2–12C mAb measured by ELISA (**D**).

Expression of human IgG in myocytes 3 d after the administration of dMAb 2–12C pDNA into the quadricep muscles of the pig confirmed the local expression of the human IgG at the site of delivery (Fig. 5A). We measured serum levels of the expressed mAb after the day 0 administration of a total dose of 24 mg dMAb 2–12C pDNA into the quadriceps of the pig. Circulating levels of the expressed mAb in the serum were detected by an HA ELISA (Fig. 5B). Peak levels were 7 and 12 μg/ml for the two animals. Influenza H1N1 neutralizing activity (1:640 peak activity in both pigs) of the serum harboring the in vivo–expressed 2–12C mAb was measured in an MN assay (Fig. 5C). In both pigs, reductions in the serum levels of the expressed mAb and serum neutralizing activity were associated with the detection of an ADA response raised in the porcine host against the human 2–12C mAb (Fig. 5B–D). These results demonstrate that dMAb 2–12C induces expression of human 2–12C after i.m. delivery by EP in mice and pigs.

### Assessment of recombinant 2–12C dosing and dMAb 2–12C in a pig influenza model

**FIGURE 6.**
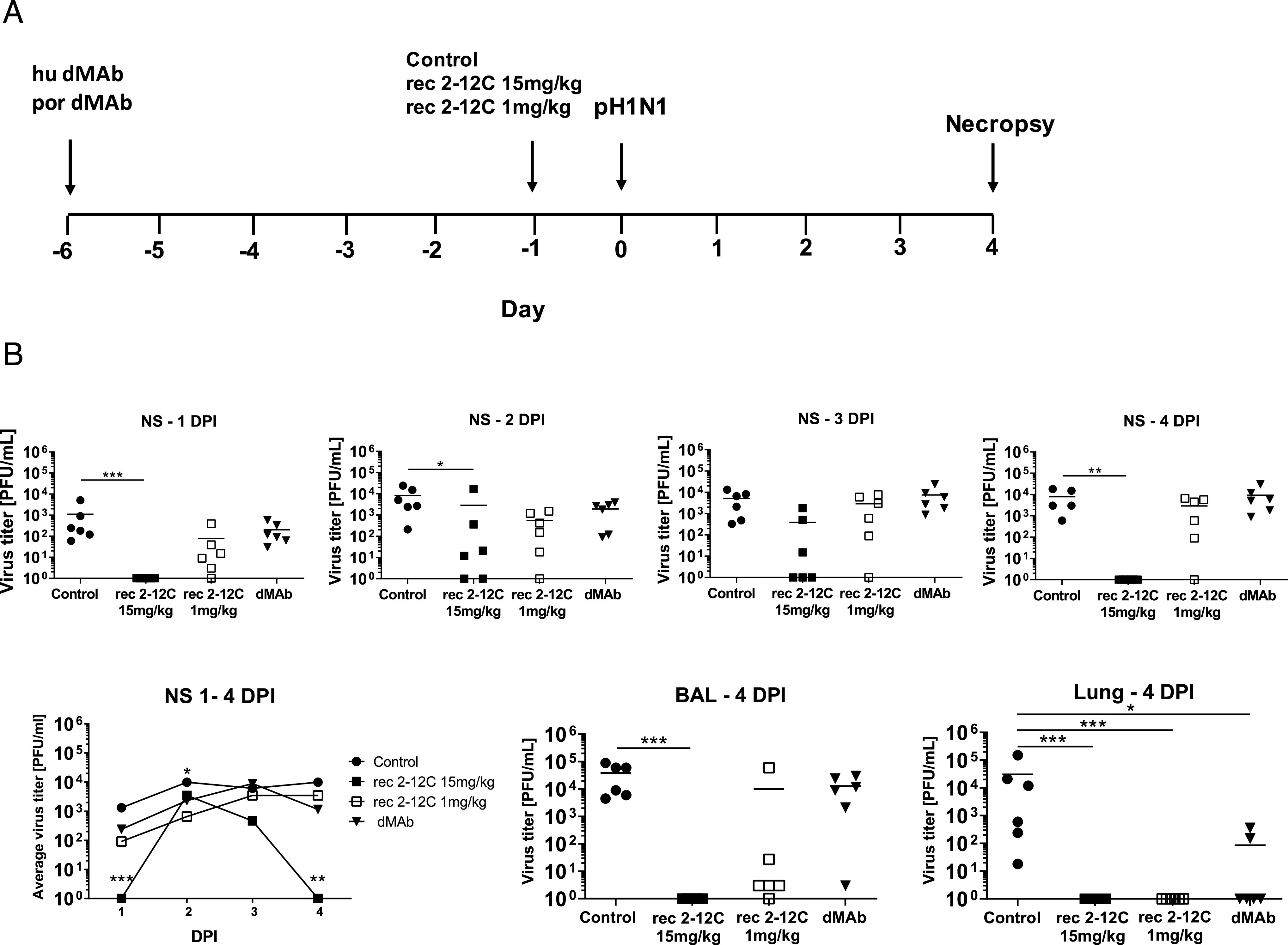
Experimental design and tissue viral load after recombinant and dMAb 2–12C treatment. Six days before pH1N1 challenge, 6 mg of dMAb 2–12C was administered i.m. by EP. Recombinant 2–12C at 15 and 1 mg/kg were delivered i.v. 24 h before challenge. Control group was untreated animals. Nasal swabs (NS) were taken at 0, 1, 2, 3, and 4 DPI, and pigs and culled at 4 DPI (**A**). Viral titers in daily NS, BAL, and accessory lung lobe (Lung) at 4 DPI were determined by plaque assay (**B**). Each data point represents an individual within the indicated group and bar is the mean. Viral shedding in NS is also shown as the mean of the six pigs over time, and significance is indicated against the control. Asterisks denote significant differences *p < 0.05, **p < 0.01, ***p < 0.001, versus control as analyzed by Kruskal–Wallis test.

After we had established 2–12C as a robust positive control Ab, we tested a lower dose of recombinant 2–12C and the efficacy of dMAb 2–12C in the pig influenza virus challenge model. The dMAb 2–12C was administered 6 d before the pH1N1 challenge to allow for accumulation of the in vivo–expressed 2–12C mAb in the host. Preliminary experiments indicated the serum levels of 2–12C were generally not negatively impacted by the ADA until day 9, so we believed this schedule allowed a window of 3–4 d to test the efficacy of dMAb 2–12C. Recombinant 2–12C at 15 and 1 mg/kg were delivered i.v. 24 h before challenge to six pigs per group (Fig. 6A).

As before, the greatest effect on virus replication in nasal swabs of recombinant 2–12C at 15 mg/kg was at day 1 and day 4 postinfection, which might be because a small number of virus particles escaped neutralization and infected only few cells so that only by days 2 and 3 has enough replication occurred to be detected. By day 4 immune mechanisms are able to control and eliminate the smaller number of infected cells.

No virus was detected in BAL and accessory lung lobe (Fig. 6B). The area under the curve in the recombinant 2–12C group was significantly different from the control (*p* = 0.0235). The lower dose of recombinant 2–12C at 1 mg/kg did not have a statistically significant effect on viral load in nasal swabs or BAL, although it reduced the titer in the lung accessory lobe. Similarly, dMAb 2–12C did not have a significant effect on viral load in nasal swabs or BAL but significantly reduced viral load in lung.

**FIGURE 7.**
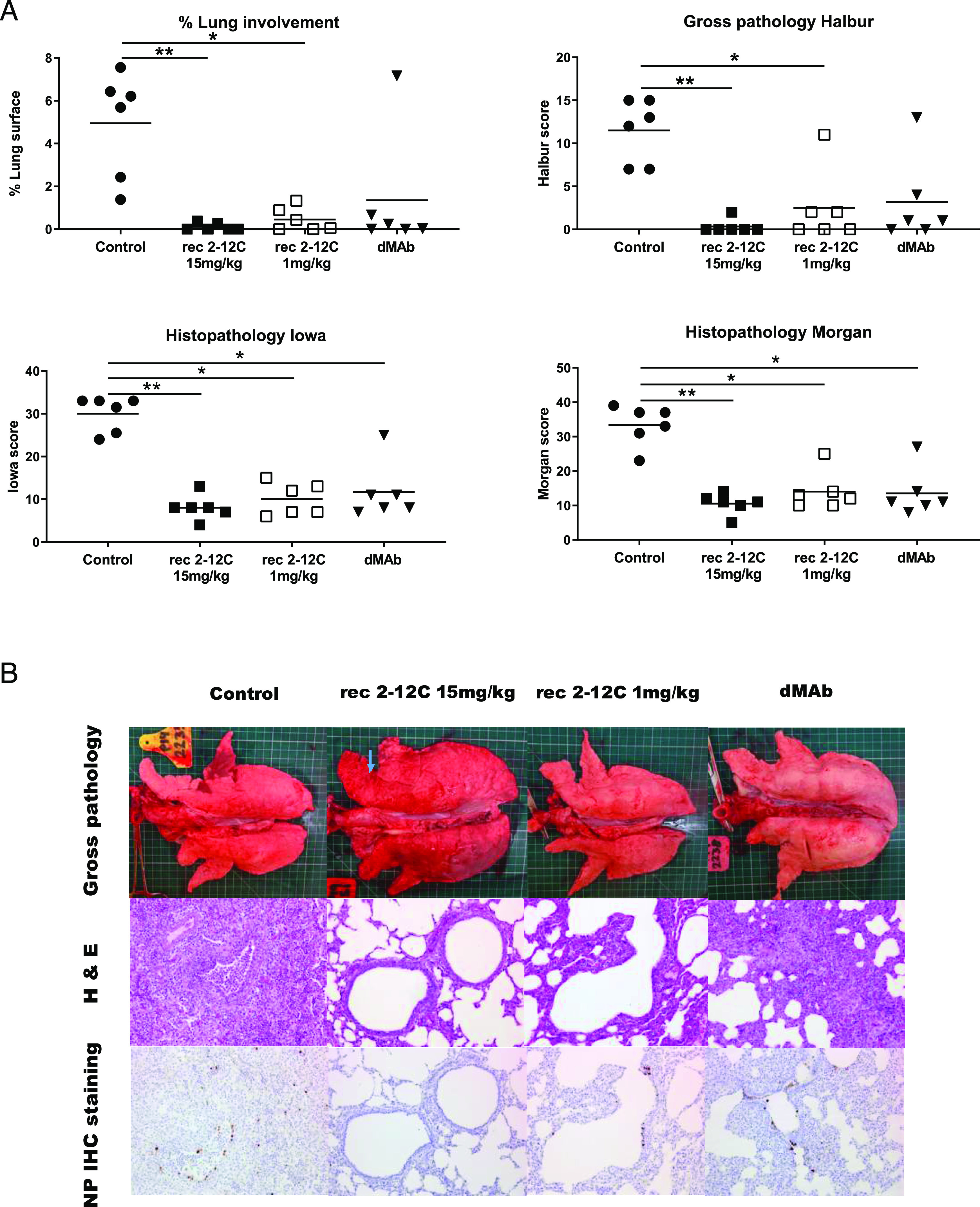
Lung pathology after recombinant and dMAb 2–12C administration. Six days before pH1N1 challenge, 6 mg dMAb 2–12C was administered i.m. by EP. Recombinant 2–12C at 15 and 1 mg/kg were delivered i.v. 24 h before challenge. Controls were untreated animals. The animals were culled at 4 DPI, and lungs were scored for appearance of gross and histopathological lesions (**A**). Representative gross pathology, histopathology (H&E staining; original magnification ×200), and immunohistochemical NP staining (original magnification ×200) for each group are shown (**B**). Asterisks denote significant differences *p < 0.05, **p < 0.01, versus control group as analyzed by Kruskal–Wallis test.

The gross pathology was reduced in all experimental groups, although this was significant only in the recombinant mAb groups because of an outlier score in one animal in the dMAb 2–12C group. Histopathological evaluation also showed significantly decreased scores in all experimental groups (Fig. 7). All animals from the control group displayed changes consistent with a mild to moderate bronchointerstitial pneumonia, with various degrees of lymphohistiocytic septal infiltration and bronchial and perivascular cuffing, and with frequent areas of necrotizing and suppurative bronchiolitis. Viral Ag was detected by IHC in bronchial and bronchiolar epithelial cells, alveolar epithelial cells, and exudate (macrophages) in all six control animals. Milder changes, mainly located in the septa, were found in animals from the Ab-treated groups. The extent of bronchiolar changes and presence of IHC labeling varied in each group, although at least one animal per group displayed bronchiolar changes and virus Ag detection, in contrast to experiment one. In addition to the differences in the histopathological scores, the recombinant 2–12C 15 mg/kg group had two animals with acute bronchial lesions and Ag detection, whereas the recombinant 2–12C 1 mg/kg group had three animals with bronchial lesions and Ag. The dMAb group had one animal with acute bronchial lesion and five animals where Ag was detected.

**FIGURE 8.**
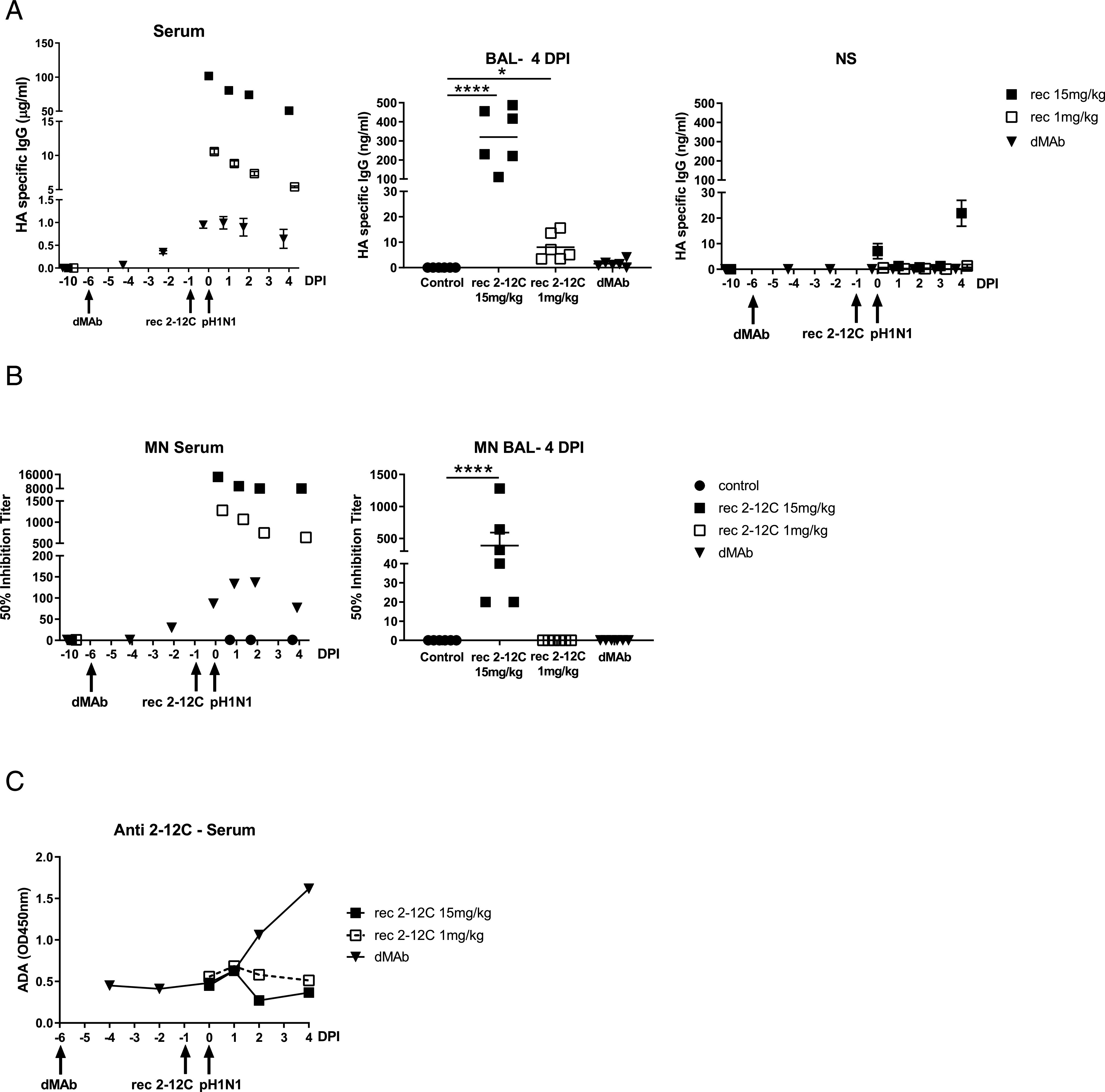
2–12C levels and antiviral activity in serum and mucosal tissues after recombinant and dMAb 2–12C administration. The concentration of 2–12C in the serum, BAL, and nasal swabs (NS) was quantified by HA-specific ELISA at the indicated time points after administration and pH1N1 challenge (**A**). The mean 50% neutralization inhibition values for the individual groups in serum over time and BAL at 4 DPI (**B**). Anti-2–12C Ab (ADA) responses in serum detected by binding to immobilized 2–12C mAb (**C**). Data were analyzed using one-way nonparametric ANOVA with the Kruskal–Wallis test. Asterisks denote significant differences ****p < 0.0001, versus control group.

The concentration of the mAbs in the serum was determined daily after challenge. The 2–12C was detected in the 15 mg/kg group at 101 μg/ml and in the 1 mg/kg group at 10 μg/ml 24 h after administration. This declined in both groups over the next 3 d (Fig. 8A). In contrast the dMAb reached its peak of 0.99 μg/ml at day 7 after administration, declining thereafter. In the BAL, HA-specific Abs were detected in the recombinant 2–12C groups (mean of 320.3 ng/ml for the 2–12C 15 mg/kg and 8 ng/ml for the 1 mg/kg 2–12C groups) and a trace in the dMAb (1.5 ng/ml). In nasal swabs, HA-specific Abs were detected in the 15 mg/kg 2–12C group at 21.9 ng/ml 4 DPI (Fig. 8A).

Neutralizing activity in serum was detected in all groups (Fig. 8B). There was 50% inhibition titer of 1:14,600 for the 15 mg/kg group and 1:1280 for the 1 mg/kg group at the time of challenge. A peak titer of 1:136 was detected at day 7 in the dMAb 2–12C-treated group. MN activity in BAL was only seen in the recombinant 2–12C 15 mg/kg group. A pig anti-2–12C ADA response was detectable in the dMAb group at day 8 after administration increasing at day 10 (Fig. 8C). No ADA response was seen to the recombinant proteins in the serum, most likely because the animals were culled only 4 d after recombinant Ab administration.

Overall, these results indicate that recombinant 2–12C offers robust protection at 15 mg/kg and that this correlates with mAb concentration and neutralization in serum. Administration of recombinant 2–12C protein at 1 mg/kg and dMAb 2–12C at 6 mg (0.5 mg DNA/kg) significantly reduced lung pathology and viral load in the lungs but not in nasal swabs or BAL.

### Sequencing of virus

Inoculant virus and viruses from nasal swab samples of two control pigs and two recombinant 2–12C (15 mg/kg)–treated pigs at 3 DPI were subjected to deep sequence analysis. In the inoculant, two control samples, and one of the recombinant 2–12C-treated samples (53_2–12C), all segments of the influenza genome had 100% coverage with lowest average per base coverage of 98,384.7 for NA segment in sample of 53_2–12C. For the other recombinant 2–12C-treated sample (51_2–12C), the lowest segment coverage was 94.8% for the NA segment with average per base coverage of 2587.7. Although the inoculant had been passed in MDCK cells for at least five times, from these sequencing data, a total of only five SNPs (three nonsynonymous SNPs) were detected as compared with the reference A/swine/England/1353/2009 (pH1N1) sequence (Table I). Nonsynonymous SNPs found in sequenced nasal swab samples of two control and two recombinant 2–12C (15 mg/kg)–treated pigs at 3 DPI compared with A/swine/England/1353/2009 are shown in Table II. One nonsynonymous SNP (HA K226E) that was detected in two control samples and one recombinant 2–12C-treated sample (51_2–12C) was also identified as one of the HA SNPs found in the inoculant virus (Table I). Three nonsynonymous SNPs were detected in the other recombinant 2–12C-treated sample (53_2–12C). One of them was also HA K226E, whereas the other 2 SNPs (HA G172E and HA T201I) were not detected in inoculant virus. Their frequencies in sample 53_2–12C are 12.1% (HA G172E) and 11.1% (HA T201I), respectively (Table II). Therefore, from our deep sequencing data, we did not see evidence of escape of virus from Ab 2–12C (15 mg/kg) at day 3. No mutations in the 2–12C binding site were detected. Similarly, no evidence for viral evolution driven by 2–12C was detected in day 4 nasal swab samples in the 1 mg/kg– and dMAb-treated groups (data not shown).

**Table I.** SNPs in inoculant virus compared with A/swine/England/1353/2009

Segment	AA Pos*^a^*	nt Pos	Ref Base	Coverage	AA Change	SNP Type	A Number	T Number	G Number	C Number	N Number*^b^*
KR701099.1_NA	454	1,361	G	106,526	GGT(G)˗GTT(V)	Nonsyn	A = 38 (0.000)	T = 18,689 (0.175)	Ref = G	C = 38 (0.000)	*n* = 0 (0.000)
KR701097.1_HA	171	511	A	281,680	AAA(K)˗GAA(E)	Nonsyn	Ref = A	T = 203 (0.001)	G = 58,387 (0.207)	C = 23 (0.000)	*n* = 0 (0.000)
KR701097.1_HA	226	676	A	104,856	AAG(K)˗GAG(E)	Nonsyn	Ref = A	T = 302 (0.003)	G = 39,918 (0.381)	C = 25 (0.000)	*n* = 0 (0.000)
KR701096.1_PA	353	1,059	G	284,452	AAG(K)˗AAA(K)	Syn	A = 41,195 (0.145)	T = 283 (0.001)	Ref = G	C = 60 (0.000)	*n* = 0 (0.000)
KR701096.1_PA	693	2,079	C	109,703	TGC(C)˗TGT(C)	Syn	A = 218 (0.002)	T = 18,512 (0.169)	G = 76 (0.001)	Ref = C	*n* = 0 (0.000)

^a^ Amino acid and nucleotide numberings start at Met or ATG.

^b^ Mixed base calls at that position.

Nonsyn, nonsynonymous; Pos, position; Ref, reference; Syn, synonymous.

**Table II.** Nonsynonymous SNPs in day 3 samples compared to A/swine/England/1353/2009

Sample	Segment	AA Pos*^a^*	nt Pos	Ref Base	Coverage	AA Change	SNP Type	A Number	T Number	G Number	C Number	N Number*^b^*	Compared with Inoculant
30_Naive	KR701097.1_HA	226	676	A	271,572	AAG(K)˗GAG(E)	Nonsyn	Ref = A	T = 45 (0.000)	G = 236,453 (0.871)	C = 81 (0.000)	*n* = 0 (0.000)	Same as inoculant
31_Naive	KR701097.1_HA	226	676	A	190,623	AAG(K)˗GAG(E)	Nonsyn	Ref = A	T = 95 (0.000)	G = 158,242 (0.830)	C = 43 (0.000)	*n* = 0 (0.000)	Same as inoculant
51_2–12c	KR701097.1_HA	226	676	A	8,398	AAG(K)˗GAG(E)	Nonsyn	Ref = A	T = 0 (0.000)	G = 8,394 (1.000)	C = 0 (0.000)	*n* = 0 (0.000)	Same as inoculant
53_2–12c	KR701097.1_HA	172	515	G	123,369	GGA(G)˗GAA(E)	Nonsyn	A = 14,937 (0.121)	T = 6 (0.000)	ref = G	C = 22 (0.000)	*n* = 0 (0.000)	New SNP
	KR701097.1_HA	201	602	C	151,699	ACT(T)˗ATT(I)	Nonsyn	A = 14 (0.000)	T = 16,907 (0.111)	G = 67 (0.000)	ref = C	*n* = 0 (0.000)	New SNP
	KR701097.1_HA	226	676	A	139,973	AAG(K)˗GAG(E)	Nonsyn	Ref = A	T = 37 (0.000)	G = 97,706 (0.698)	C = 54 (0.000)	*n* = 0 (0.000)	Same as inoculant

^a^ Amino acid and nucleotide numberings start at Met or ATG.

^b^ Mixed base calls at that position.

Nonsyn, nonsynonymous; Pos, position; Ref, reference; Syn, synonymous.

## Discussion

In the last decade there has been extensive research on the use of mAbs for passive immunization against influenza. mAbs could be used as pre- or postexposure treatment to prevent or reduce severe disease. They have the advantage of providing immediate immunity and bridging the gap between the start of a pandemic and vaccine availability. To test candidate mAbs and delivery platforms, we have established a reproducible and robust pig influenza challenge model and identified a protective human HA1-specific mAb, 2–12C, which can be used as a standard to benchmark other mAb candidates and delivery platforms. Both viral load and pathology were significantly reduced when the recombinant 2–12C mAb was given at 15 mg/kg i.v. 24 h before pH1N1 challenge. Furthermore, the mAb-mediated a significant reduction in lung pathology upon administration at a lower dose as a recombinant protein (1 mg/kg) or as a dMAb (0.5 mg DNA/kg). No evidence for 2–12C-driven viral evolution was detected in any group. However, it is important to reflect that 2–12C, which is a typical mAb to the globular head of HA, selects resistance mutations in vitro at position K130 (31, 44). Although the epitope recognized by 2–12C has remained stable in circulating seasonal H1N1 viruses for 10 y, in 2019, viruses have appeared with a substitution at N129D in the HA that are resistant to neutralization by 2–12C (Crick Reports September 2019, https://www.crick.ac.uk/partnerships/worldwide-influenza-centre/annual-and-interim-reports). Although Abs to the globular head are profoundly protective, they will need to be regularly updated in contrast to broadly reactive anti-stem HA Abs.

Conventional mAbs remain an expensive approach from a manufacturing perspective so that a simple, cost-effective passive immunization strategy inducing sustained in vivo production would be extremely valuable. dMAbs, have the potential to circumvent cost constraints and provide durable immunity, perhaps for the duration of an influenza pandemic or season (24–27). In this study, we tested 2–12C dMAb in the pig influenza model and although the serum concentration of in vivo–expressed dMAb was 100 times lower (∼1 μg/ml) than in pigs given the recombinant protein at 15 mg/kg (∼100 μg/ml), we observed significant protection against disease pathology in the lungs. This was encouraging as a relatively low dose of dMAb was used (0.5 mg DNA/kg), and the anti-human 2–12C response induced in the pig prevented peak concentration from being achieved (Figs. 5, 6).

We also tested 2–12C dMAb, partially porcinised with pig Fc IgG3, and observed similar protection and an ADA response (data not shown), indicating that the human Fab still induces an Ab response. Fig. 4D exemplifies a typical pharmacokinetic curve in the absence of an ADA. We predict using species-matched Abs will circumvent this barrier in pigs. Of note, ongoing studies in sheep using dMAbs based on bovine Abs have shown durable circulating mAb levels in the absence of ADA responses (K. Hollevoet, T.R.F. Smith, K. Schultheis, N. Geukens, and K.E. Broderick, manuscript in preparation). We have generated a number of high affinity pig influenza virus–neutralizing Abs that will provide a more relevant test system to evaluate delivery of novel dMAbs and other emerging platforms in pigs. Furthermore, these porcine mAbs will allow detailed investigation of pharmacokinetics and protective mechanisms in a relevant natural large host animal model.

In our previous studies we used the human broadly neutralizing anti-stem FI6 mAb and showed only marginal protective effect on gross pathology after aerosol delivery. We were unable to detect binding of FI6 to porcine PBMC or Ab-dependent cellular cytotoxicity when porcine PBMCs were used. More refined analysis of binding of human IgG to the Göttingen minipig Fc γ receptors (FcγR) indicated that the binding affinities for human IgG to porcine FcγRIa, FcγRIIa, and FcγRIIb were comparable to the respective human FcγRs (45). However, there was no binding of hu IgG1 to the poFcγRIIIa, which is an important mediator of Ab-dependent cellular cytotoxicity in monocytes and NK cells. The lack of binding to porcine poFcγRIIIa may abrogate or greatly reduce Fc-mediated functions of human mAbs in agreement with our previous study (28). This data suggest that there is a need for further investigation of which Fc receptors, Ig classes, and Ig subclasses are critical for therapeutic effects of mAbs in vivo.

An essential component in development of mAbs therapies is the need to improve existing animal models to more closely mimic humans. Unfortunately, all animal models have limitations in recapitulating the full range of disease observed in humans. Mice, guinea pigs, ferrets, and nonhuman primates are used for influenza virus research, with the ferret considered to represent the “gold standard.” However, mice cannot be infected with most strains of the influenza virus and do not recapitulate signs of illness observed in humans, guinea pigs do not exhibit overt signs of illness, and ferrets may have different drug pharmacokinetics to humans (46–49). Nonhuman primates share many physiological and genetic similarities to humans and are susceptible to influenza virus infection, although they show symptoms only after intratracheal challenge with a high-pathogenic virus. Furthermore, there are ethical considerations, they are costly, not easily accessible, and generally weigh as little as 2–4 kg. In contrast, the pig is a large animal and a natural host for the same subtypes as human seasonal strains as well as a source of new human pandemic viruses (16). Our pigs were between 11 and 14 kg, but pigs with equivalent weights to humans are available. A further advantage of the pig model is that the same pdmH1N1 virus circulates in both pigs and humans and has been used in human challenges as well as in our pig model (50–52). Therefore, findings in the pig could be directly tested in humans to further validate the model.

In summary we have established a positive control protective influenza mAb and delivery method to benchmark mAb delivery platforms. This will enable testing of further improvements in the mAb delivery platforms, the effect of therapeutic administration, and whether mixtures of mAbs will provide synergy by harnessing both classical neutralization and Fc-mediated effector mechanisms. Furthermore, research in the pig has the additional benefit in facilitating development of new platforms for interventions in livestock diseases. We propose that the pig influenza model will become a critical tool to accelerate mAb development to the clinic by validating lead mAbs from smaller animal models and evaluating emerging delivery approaches.
